# Chronic Periodontitis and Alzheimer Disease: A Putative Link of Serum Proteins Identification by 2D-DIGE Proteomics

**DOI:** 10.3389/fnagi.2020.00248

**Published:** 2020-08-21

**Authors:** Xianfang Rong, Liping Xiang, Yanfen Li, Hongfa Yang, Weijian Chen, Lei Li, Defeng Liang, Xincai Zhou

**Affiliations:** ^1^Department of Stomatology, Shenzhen Baoan Women’s and Children’s Hospital, Jinan University, Shenzhen, China; ^2^Department of Cardiology, The Second Affiliated Hospital of the University of South China, Hengyang, China

**Keywords:** chronic periodontitis, Alzheimer’s disease, two-dimensional differential in-gel electrophoresis, biomarker, Cathepsin B

## Abstract

Increasing evidence indicates Chronic Periodontitis (CP) is a comorbidity of Alzheimer’s disease (AD), which is the most common form of age-related dementia, and for the latter, effective diagnostic and treatment strategies are lacking. Although inflammation is present in both diseases, the exact mechanisms and cross-links between CP and AD are poorly understood; and a direct association between the two has not been reported. This study aimed to identify a direct serum proteins link between AD and CP. Two-dimensional differential in-gel electrophoresis was employed to analyze serum samples from 12 CP patients and 12 age-matched controls. Furthermore, to determine the molecular link between CP and AD, neuroblastoma SK-N-SH APPwt cells were treated with 1 μg/ml of lipopolysaccharide from *Porphyromonas gingivalis* (P.g-LPS). Ten differentially expressed proteins were identified in CP patients. Among them, nine proteins were up-regulated, and one protein was down-regulated. Of the 10 differentially expressed proteins, five proteins were reportedly involved in the pathology of AD: Cofilin-2, Cathepsin B, Clusterin, Triosephosphate isomerase, and inter-alpha-trypsin inhibitor heavy chain H4 (ITI-H4). Western blotting indicated significantly higher expression of Cofilin-2, Cathepsin B, and Clusterin and lower expression of ITI-H4 in the CP group than in the Control group. The serum concentration of Cathepsin B has a good correlation with MMSE scores. Moreover, the protein level of Cathepsin B (but not that of ADAM10 and BACE1) increased significantly along with a prominent increase in Aβ_1–40_ and Aβ_1–42_ in the cell lysates of P.g-LPS-treated SK-N-SH APPwt cells. Cathepsin B inhibition resulted in a sharp decrease in Aβ_1–40_ and Aβ_1–42_ in the cell lysates. Furthermore, TNF-α was one of the most important inflammatory cytokines for the P.g-LPS-induced Cathepsin B upregulation in SK-N-SH APPwt cells. These results show that CP and AD share an association, while Cathepsin B could be a key link between the two diseases. The discovery of the identical serum proteins provides a potential mechanism underlying the increased risk of AD in CP patients, which could be critical for elucidating the pathophysiology of AD.

## Introduction

Alzheimer’s disease (AD) is one of the most common forms of dementia among elderly people and is pathologically characterized by senile plaques and neuro-fibrillary tangles and clinically characterized by progressive deterioration of episodic memory and cognitive decline (Hardy and Higgins, 1992). The acknowledged history of AD spans almost a century, starting with its first description by Alois Alzheimer in 1907 (Alzheimer et al., 1995). During recent decades, the sequential events that occur in the development and pathology of AD have been extensively studied, yet the precise etiology of the disease remains elusive, and current preventative and curative strategies are largely unsuccessful (Rong et al., 2017; Rasmussen and Langerman, 2019). Considering the complexity of AD pathology, it is apparent that other risk factors also exist besides those known, such as gender, education, smoking, dietary habit, depression, hypertension, diabetes mellitus, obesity, and head injury (Shinohara et al., 2014; Dursun et al., 2016; Badea et al., 2019; Jackson et al., 2019; Tapiainen et al., 2020).

Chronic Periodontitis (CP) is an oral chronic infection/inflammatory disease that affects a considerable worldwide population (López and Baelum, 2015). CP comprises both gingivitis and periodontitis; for the latter, inflammation is localized in the gingival tissues or the inflammatory process reaches deeper connective and bone tissue, causing bone and attachment loss that may ultimately lead to tooth loss (Lang et al., 2009). This local inflammatory process may induce a systemic inflammatory state *via* mechanisms including dissemination of pro-inflammatory cytokines or bacteria or both from oral to extra-oral sites or even to blood circulation, which may contribute to the exacerbation of several diseases (Martins et al., 2016).

The following two findings support that comorbidity exists between CP and AD: first, AD patients have greater impairment of oral health because of their progressive cognitive impairment, which affects their oral hygiene habits; second, chronic CP can trigger or exacerbate the neuro-inflammatory process observed in AD (Kamer et al., 2015; Pazos et al., 2018). However, interventional studies reporting a direct association between CP and AD are still lacking (Teixeira et al., 2017; Olsen and Singhrao, 2020).

The vascular channel is reported to be the primary link between oral bacteria or pro-inflammatory molecules and the brain (Balan et al., 2011; Maurer et al., 2018). Accordingly, the serum is the preferred specimen for the study of comorbidity of the two diseases. Approximately 500 ml of cerebrospinal fluid is absorbed into the blood daily; thus, the serum may offer a rich source of brain-related disease biomarkers (Asgari et al., 2015). As an improvement of 2D-PAGE, two-dimensional differential in-gel electrophoresis (2D-DIGE) provides a novel opportunity to identify biomarkers or therapeutic targets (Murphy and Dowling, 2018). DIGE incorporates three types of fluorescent molecules (CyDyes), which are used to pre-label samples before separation by 2-DIGE. Proteins of interest are identified by tandem mass spectrometry [liquid chromatography-mass spectrometry/mass spectrometry (LC-MS/MS); Pasquali et al., 2017].

In this study, we aimed to: (i) identify and validate differentially expressed proteins in CP patients and controls (*n* = 23 for CP patients; *n* = 45 for age-matched healthy controls); and (ii) explore the molecular mechanism of Cathepsin B as a link between CP and AD *in vitro*.

## Materials and Methods

### Human Serum Sample Collection

The study was approved by the Medical Ethics Committee of Shenzhen Baoan Women’s and Children’s Hospital and all subjects signed informed consent before enrolment in the study. Detailed demographic information of CP patients and age-matched controls are presented in Table 1. A serum sample from 23 CP patients and 45 age-matched controls were collected. Blood samples were collected into evacuated collection tubes with no anticoagulant and were allowed to clot for 2 h on ice before centrifugation at 3,000 g for 10 min and 4°C. Serum was collected and stored in Eppendorf tubes at −80°C until utilized for the study.

**Table 1:** Characteristics of Chronic Periodontal disease (CP) patients and control subjects.

	Subjects	
	Control	CP
Numbers	45	23
Mean age (years)	62.5 ± 3.4	63.2 ± 4.1
Sex, M/F	21/24	10/13
Mean MMSE score	27.8 ± 1.6	20.4 ± 3.3
Mean duration of disease (years)		10.8 ± 0.5

*MMSE, Mini-Mental State Examination; Data are displayed as the mean ± SEM*.

### Serum Pre-fractionation

The process of depletion and desalination was performed according to the protocol described previously. The Agilent Human 14 Multiple Affinity Removal Column (Hu-14, 4.6 × 50 mm) was used to remove the most abundant proteins from the serum, which include IgG, IgA, IgM, albumin, antitrypsin, transthyretin, haptoglobin, transferrin, fibrinogen, alpha1-acid glycoprotein, alpha2-macroglobulin, apolipoproteinAII, apolipoprotein AI and complement C3. After depletion, the centrifugal concentrators (YM-3, MWCO3 kDa, Millipore) were used to desalt and concentrate the samples (Lu et al., 2014).

### 2D-DIGE Analysis and Image Analysis

The standard of 2D-DIGE analysis was performed according to the protocol described previously (Dowling and Ohlendieck, 2018). Most importantly, to reduce the variation of signal in gels, the photomultiplier tube (PMT) was set to ensure maximum pixel intensity values for all gel images within a range of 40,000–60,000 pixels. DeCyder 7.0 (GE Healthcare) was used to analyze the images. In the DIA module, each spot was detected, matched, and normalized; in the BVA module, spot statistics were reviewed. The spots with an average ratio of more than +1.5 or less than −1.5 and with a statistical difference (*p* < 0.05) were isolated for further investigation.

### Identification of Candidate Protein Biomarkers by LC-MS/MS

The standard LC-MS/MS and database searching were performed according to the protocol described previously (Sun et al., 2015). Briefly, LC-MS analysis was carried out using a Surveyor MS Pump Plus HPLC system coupled to a Thermo Fisher Finnigan LTQ linear ion trap mass spectrometer (Thermo Fisher Corporation, San Jose, CA, USA) using nano-electrospray ionization. Tryptic peptides were loaded onto a trap column (300SB-C18, 5 × 0.3 mm, 5 μm particle size; Agilent Technologies, Santa Clara, CA, USA) connected through a zero dead volume union to the self-packed analytical column (C18, 100 × 0.1 mm, 3 μm particle size; SunChrom, Germany). The peptides were then separated by linear gradient elution involving 0–45% B over 55 min followed by 45–100% B over 10 min (B is 80% acetonitrile, 0.1% formic acid) at a flow rate of 500 nL/min. MS data were analyzed using SEQUEST against the National Center for Biotechnology Information (NCBI) human protein database and the results filtered, sorted, and displayed using Bioworks 3.2. Returned protein lists were filtered using the parameters: Peptide Xcorr value >1.90 (for +1 charge), >2.75 (for +2 charge), >3.75 (for +3 charge); peptide Delt CN >0.1; protein probabilities <0.001. At least two unique peptides were required for each identified protein.

### Western Blot Analysis

Western blot was performed as described before (Sun et al., 2015). Briefly, the PVDF membranes were incubated with anti-Cofilin-2 (Santa Cruz, sc-166985), anti-Cathepsin B (Cell Signal Technology, 31718), anti-Triosephosphate isomerase (Abcam, ab28760), anti-Clusterin (CST, 34642), anti-ITI-H4 (Santa Cruz, sc-515353), anti-APP (Cell Signal Technology, 29765), Anti-sAPPα (IBL, 11088), Anti-sAPPβ (IBL, 18957) Anti-BACE1 (Abcam, ab2077), anti-ADAM10 (Cell Signal Technology, 14194) and anti-β-actin (Cell Signal Technology, 3700) overnight at 4°C. After washed with TBST, HRP-conjugated secondary antibodies (1:10,000) were applied at room temperature for 1 h. The signals were detected by a ChemiDoc MP system (Bio-Rad) and analyzed by ImageJ software.

### Enzyme-Linked Immunosorbent Assay (ELISA)

ELISA was performed according to the instruction of each kit: Human Amyloid beta (aa1–40) ELISA Kit (DAB140B, R&D Systems), Human Amyloid beta (aa1–42) ELISA Kit (DAB142, R&D Systems) and Human Cathepsin B ELISA Kit (ab119584, Abcam). Each sample was performed in duplicate. Briefly, Serum (or diluted serum) at 100 μl was added to the plate and incubated for 2 h at 2–8°C. After a total of four washes, Conjugate was added and incubated for another 2 h at 2–8°C. After another four washes, Substrate Solution was added to each well and incubated for 30 min at room temperature. For signal detection, each well was determined using 450 nm as a primary wavelength and 630 nm as a reference wavelength.

### Cell Culture

Human neuroblastoma SK-N-SH cells overexpressing wild-type APP695 (SK-N-SH APPwt) were a gift from Dr. Dennis Selkoe (Boston, MA, USA). SK-N-SH APPwt cells were grown in DMEM, plus10% fetal bovine serum (Hyclone, Los Angeles, CA, USA), 100 U/ml penicillin/streptomycin. Also, cells were supplemented with 200 μg/ml G418. Cell cultures were maintained at 37°C in a humidified atmosphere containing 5% CO2 and passed every 2–4 days based on 85% confluence. P.g-LPS was obtained from Invivo Gen (San Diego, CA, USA), and applied to the supernatant of SK-N-SH APPwt cells with the final concentration 1 μg/ml for a total of 7 days to mimic CP *in vitro* (P.g-LPS was incubated for 4 days initially. At confluence, SK-N-SH APPwt cells were passaged and new P.g-LPS were applied to the supernatant immediately and incubated for another 3 days). For Cathepsin B inhibition, a final concentration of 75 μM CA-074 methyl ester (Sigma-Aldrich, USA) was applied to the supernatant of SK-N-SH APPwt cells 1 h before P.g-LPS. For Cathepsin B activation, IL-6 (10 ng/ml, Genscript, Z03134), IL-1β(100 pg/ml, Genscript, Z02978), TNF-α (10 ng/ml, Genscript, Z01001) and recombinant Human CRP protein (1 mg/L, Abcam ab171471) were applied to SK-N-SH APPwt cells for 24 h. For TNF-α inhibition, pomalidomide (2 μM, Selleck, S1567) was applied to P.g-LPS treated SK-N-SH APPwt cells 24 h before cell harvest.

### Statistical Analysis

For the 2D-DIGE experiment study, DeCyder 7.0 (GE Healthcare) was used to analysis data from DIGE (DIA and BVA model). Spots with *p* < 0.05 and variation ratio >1.5-fold between groups were considered as the differential spots. For Western blot data, comparison between the groups was made using a two-tailed unpaired Student’s *t*-test. The Control group was normalized to 100%. All data were shown as mean ± SD, and prism software (GraphPad Prism5, La Jolla, CA, USA) was used to create the graphs. A value of *p* < 0.05 was considered to be statistically significant. The correlation between Cathepsin B level and MMSE scores were performed with the Spearman correlation coefficient.

## Results

### Clinical Data

The clinical data from each subject are summarized in Table 1. No significant differences were found in age, sex, and education between the two groups in either the 2-DIGE study or the validation study (*p* > 0.05).

### Serum Aβ Measurements in the Clinical Cohort

**Figure 1:**
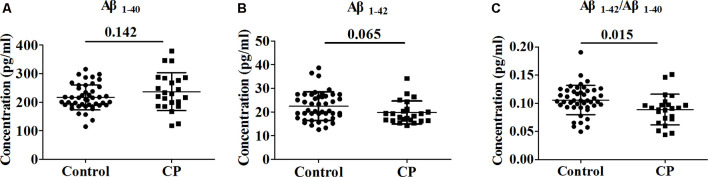
Comparison of Aβ_1–40_, Aβ_1–42_ and Aβ_1–42_/Aβ_1–40_ between Chronic Periodontitis (CP) group and Control group. **(A)** The concentration of Aβ_1–40_ between CP group and Control group. **(B)** The concentration of between CP group and Control group. **(C)** The concentration of Aβ_1–42_/Aβ_1–40_ between CP group and Control group. No significant difference between the CP group and Control group in Aβ_1–40_ (*p* = 0.142), Aβ_1–42_ (*p* = 0.065) were observed. Aβ_1–42_/Aβ_1–40_ was significantly decreased in the CP group compared to the Control group (*p* = 0.009).

Whole serum from the CP group and Control group were analyzed to determine the concentration of Aβ_1–40_ and Aβ_1–42_. The serum levels of Aβ_1–40_ and Aβ_1–42_ did not differ significantly between the groups, with observed serum levels of 216.9 ± 44.0 and 237.0 ± 66.6 pg/ml for Aβ_1–40_ in the CP and Control groups, respectively (*p* = 0.142) and 22.5 ± 6.0 and 19.8 ± 4.8 pg/ml for Aβ_1–42_ in the CP group and Control group, respectively (*p* = 0.065). However, the Aβ_1–42_/Aβ_1–40_ ratio was notably lower (*p* = 0.009) in the CP group than in the Control group, with serum levels of 0.1057 ± 0.0258 and 0.0889 ± 0.0271 pg/ml, respectively (Figure 1).

### Identification of Differentially Expressed Proteins in CP Patients Using 2D-DIGE

**Figure 2:**
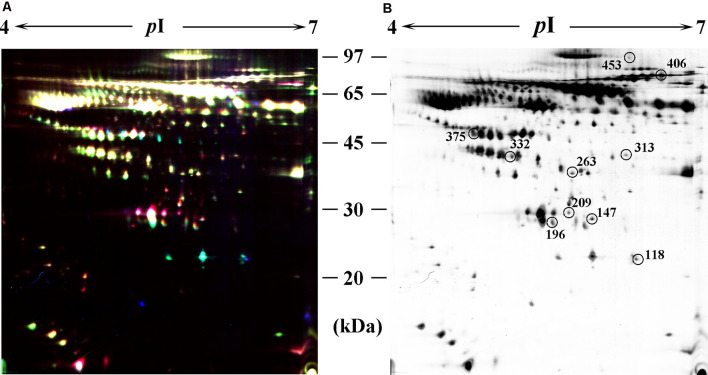
Identification of differentially expressed spots by using two-dimensional differential in-gel electrophoresis (2D-DIGE). Abundant protein depleted serum was subjected to 2D-DIGE quantitative analysis to identify proteins with differing abundance between the CP group and Control group. **(A)** Over-lay of cy2-marked loading control, cy3-marked, and cy5-marked serum sample from CP patient and age-matched controls (*n* = 12). **(B)** Distribution of differentially expressed protein spots. The samples were separated using IPG gel (pH 4–7, 18 cm) in the first phase and 12.5% SDS-PAGE; 150 ug of protein was used in each gel. The spots showing significant differences between CP patients and controls (see Table 2) were labeled in a 2D-DIGE gel.

In the differential in-gel analysis (DIA) workspace, approximately500 spots were detected in each gel by the Decyder software. In the Biological variation analysis (BVA) module, the Cy2 image from gel number five was selected as the master gel, as it had the maximum number of spots. Overall, 10 spots were found to be differentially expressed with the criteria (Figure 2B). The 10 spots of interest were manually excised from colloidal Coomassie-stained preparative gels of the pooled CP patients’ and age-matched controls’ depleted serum for in-gel trypsin proteolysis and subsequent LC-MS/MS (LTQ) analysis (Table 2). All 10 differentially expressed protein spots corresponded to 10 different proteins; Cofilin-2 (118 spots), Heat shock protein beta-1 (147 spots), Retinol-binding protein 4 (196 spots), Triosephosphate isomerase (209 spots), Cathepsin B (263 spots), Haptoglobin (313 spots), Alpha-1-antitrypsin (332 spots), Clusterin(375 spots), Complement factor B (406 spots), and inter-alpha-trypsin inhibitor heavy chain H4 (ITI-H4; 453 spots). These proteins were consistent with the theoretical molecular weights and *p*I ranges based on the positions of the spots on the gel.

**Table 2:** Differentially expressed serum proteins in CP patients as compared with controls.

Spot No.^a^	Protein definition	Accession GI No.	Score	Sequence coverage (%)	p*I*^b^	MW^c^	Variation ratio	*p*-value
118	Cofilin-2	6671746	110.26	33.9	6.77	18709.5	+9.46	0.0014
147	Heat shock protein beta-1	4504517	40.20	11.7	5.98	22782.5	+5.23	0.0064
196	Retinol-binding protein 4	18088326	63.16	33.7	5.76	23010.0	+5.45	0.0011
209	Triosephosphate isomerase	1906326	170.29	57.1	5.65	30791.7	+25.37	0.0354
263	Cathepsin B	6681089	50.39	76.1	5.88	37821.6	+10,000	0.0217
313	Haptoglobin	292156	30.27	15.9	6.13	45861.5	+5.20	0.0016
332	Alpha-1-antitrypsin	189163542	150.21	61.4	5.37	46736.5	+2.27	0.0004
375	Clusterin	355594753	80.21	22.5	4.68	52494.5	+2.63	0.0434
406	Complement factor B	4261689	110.63	36.5	6.67	85532.9	+10,000	0.0059
453	ITI-H4	262050538	80.26	23.2	6.53	103358.1	−43.52	0.0036

*Serum proteins from CP patients and controls were separated by DIGE and compared DeCyder 7.0 (GE Healthcare). Spots with *p* < 0.05 and variation ratio >1.5-fold between groups were considered as the differential spots. “Ratio = 10,000” means the spot presented only in one group. Their identities were determined by LC-MS/MS as described in “Materials and Methods” section. Spot numbers correspond to those in Figure 2. ^a^Spot numbers correspond to those in Figure 2B. ^b^pI is the theoretical calculated from the amino acid sequence of the predicted mature protein. ^c^MW refers to the theoretical molecular mass is kDa calculated from the amino acid sequence*.

### Protein Validation of the Differential Proteins Using Whole Serum

**Figure 3:**
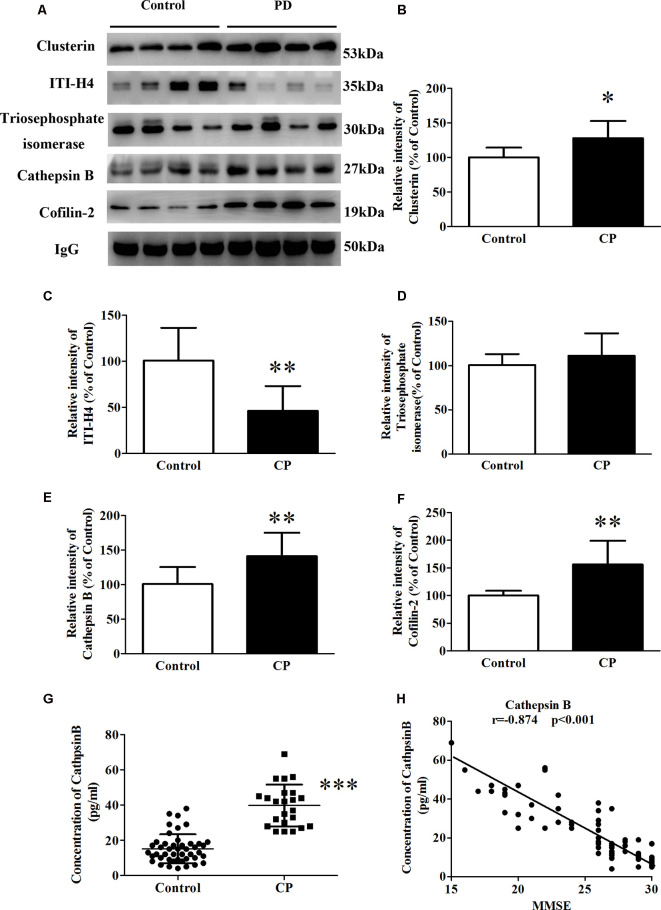
The expression of Clusterin, ITI-H4, Triosephosphate isomerase, Cathepsin B, and Cofilin-2 between Control subjects and CP patients. **(A)** A representative panel of Western blots of Clusterin, ITI-H4, Triosephosphate isomerase, Cathepsin B and Cofilin-2. **(B)** Quantitative comparison of the Western blot of Clusterin. **(C)** Quantitative comparison of the Western blot of ITI-H4. **(D)** Quantitative comparison of the Western blot of Triosephosphate isomerase. **(E)** Quantitative comparison of the Western blot of Cathepsin B. **(F)** Quantitative comparison of the Western blot of Cofilin-2. **(G)** The serum concentration of Cathepsin B for each individual was measured by ELISA. **(H)** The correlation between serum Cathepsin B and MMSE score. Data represent mean ± SEM for 16 individual subjects per group. *p < 0.05 compared with the Control group, **p < 0.01 compared with the Control group, ***p < 0.001 compared with Control group, student’s *t*-test.

Whole serum was used in the validation study by western blot analysis, in line with clinical practice. In the validation study, five differential proteins were selected for validation by western blot analysis because of their well-established relationship with AD (Figure 3A). In line with the 2D-DIGE study, the expression of Cofilin-2, Cathepsin B, and Clusterin was respectively increased by 58.3, 42.6, and 27.5% (Figures 3B,E,F), whereas the expression of ITI-H4 was reduced by 60.8% (Figure 3C) in whole serum from the CP group. In contrast to the 2D-DIGE results, the expression of Triosephosphate isomerase remained unchanged (Figure 3D). Cathepsin B was selected for further validation by ELISA. The results showed a huge increase of Cathepsin B serum concentration in CP patients (39.7 ± 2.5) as compared to controls (15.2 ± 1.2; Figure 3G). The serum concentration of Cathepsin B correlated well with MMSE scores (*r* = −0.874, *p* < 0.001; Figure 3H).

### Cathepsin B in SK-N-SH APPwt Cells

**Figure 4:**
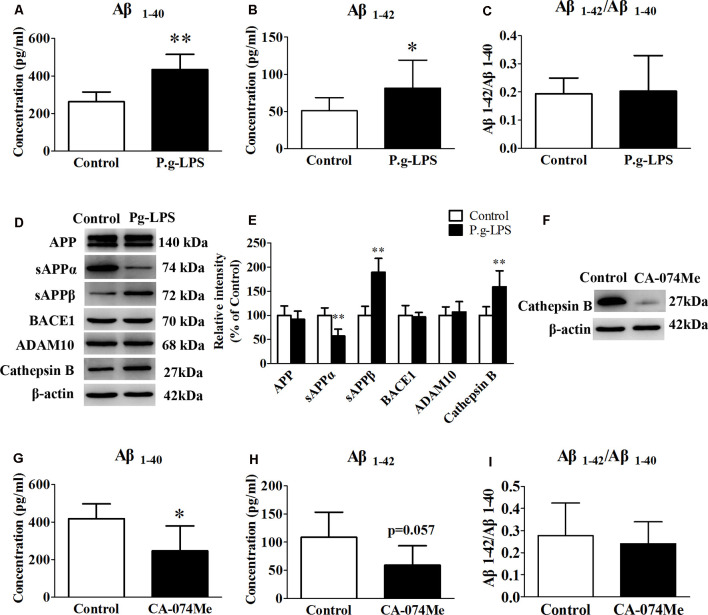
Cathepsin B inhibition could decrease the concentration of Aβ_1–40_ and Aβ_1–42_ in P.g-LPS-treated SK-N-SH APPwt cells. **(A–C)** Aβ_1–40_, Aβ_1–42_, Aβ_1–42_/Aβ_1–40_ concentration in SK-N-SH APPwt cells and P.g-LPS-treated SK-N-SH APPwt cells. **(D,E)** Representative panel and Quantitative comparison of the Western blot of APP, sAPPα, sAPPβ, BACE1, ADAM10, and Cathepsin B in the cell lysates of SK-N-SH APPwt cells and P.g-LPS-treated SK-N-SH APPwt cells. **(F)** A representative panel of the Western blot of Cathepsin B in the cell lysates of P.g-LPS-treated SK-N-SH APPwt cells after CA-074Me application. **(G–I)** Aβ_1–40_, Aβ_1–42_ and Aβ_1–42_/Aβ_1–40_ concentration after Cathepsin B inhibition in P.g-LPS-treated SK-N-SH APPwt cells. ^ *^p < 0.05 compared with Control group, **p < 0.01 compared with Control group, student’s *t*-test.

To determine the molecular link between CP and AD, neuroblastoma SK-N-SH APPwt cells were treated with 1 μg/ml of P.g-LPS for 7 days. Results show that P.g-LPS treatment significantly increased both the Aβ_1–40_ (*p* < 0.01) and Aβ_1–42_ (*p* < 0.05) levels in the cell lysates of SK-N-SH APPwt cells (Figures 4A–C). To identify the underlying mechanism, we investigated the effect of P.g-LPS on the APP-processing enzymes ADAM10, BACE1, and Cathepsin B and APP cleavage fragments using western blotting. P.g-LPS treatment significantly increased the protein level of sAPPβ (*p* < 0.01) and Cathepsin B (*p* < 0.01), with a considerable decrease of sAPPα (*p* < 0.01; Figures 4D,E). To further verify our results, a specific Cathepsin B inhibitor CA074Me was applied to assess Cathepsin B inhibition on the Aβ_1–40_ and Aβ_1–42_ levels in SK-N-SH APPwt cells. The results indicated a robust decrease in the Aβ_1–40_ (*p* < 0.05) and Aβ_1–42_ levels in P.g-LPS-treated-SK-N-SH APPwt cells after Cathepsin B inhibition (Figures 4G–I).

### Inflammatory Cytokines in P.g-LPS Treated SK-N-SH APPwt Cells

**Figure 5:**
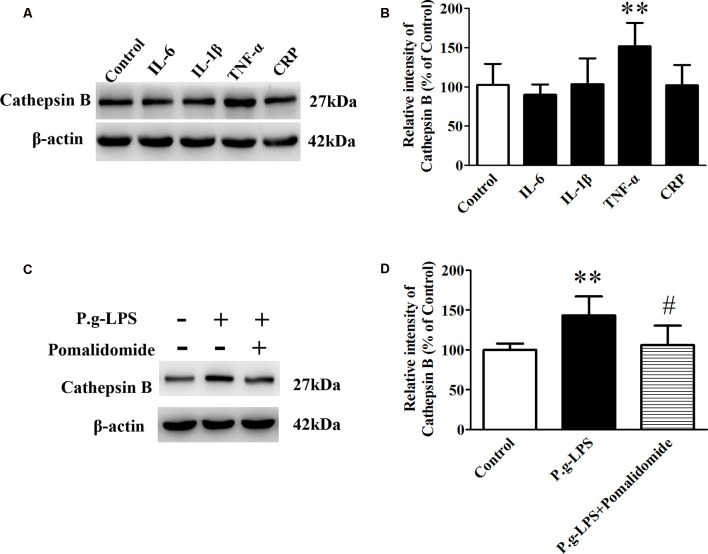
TNF-α is responsible for the increase of Cathepsin B in P.g-LPS-treated SK-N-SH APPwt cells. **(A,B)** Representative panel and quantitative comparison of the Western blot of Cathepsin B in IL-6-treated group, IL-1β-treated group, TNF-α-treated group, and recombinant Human CRP-treated group and Control group. **(C,D)** Representative panel and quantitative comparison of the Western blot of cathepsin B in Control group, P.g-LPS-treated group, and P.g-LPS plus pomalidomide-treated group. ^#^*p* < 0.05 compared to P.g-LPS group, **p < 0.01 compared with Control group, student’s *t*-test.

To elucidate the underlying mechanism of P.g-LPS-induced Cathepsin B up-regulation in SK-N-SH APPwt cells, the well-known inflammatory cytokines in CP including IL-6, IL-1β, and TNF-α, and CRP were applied to the supernatant of SK-N-SH APPwt cells. Only TNF-α treatment could significantly raise the protein level of Cathepsin B (*p* < 0.01; Figures 5A,B). Furthermore, pomalidomide, a specific TNF-α inhibitor was applied. The results showed in the context of TNF-α inhibition, P.g-LPS could not up-regulate the protein expression of Cathepsin B, which confirmed it was TNF-α which was responsible for P.g-LPS induced Cathepsin B up-regulation in SK-N-SH APPwt cells (Figures 5C,D).

## Discussion

Several studies have indicated that patients with AD have poorer dental health than age-matched controls, while other studies have also confirmed that patients with CP have cognitive defects and even dementia (Pritchard et al., 2017; Dominy et al., 2019). However, the mechanism underlying the relationship between periodontitis and cognitive decline remains unclear (Gaur and Agnihotri, 2015; Chen et al., 2017). In this study, for the first time, we examined the correlation between CP and AD, regarding proteomics.

Approximately 500 protein spots were successfully matched between the two groups on each gel, and 10 proteins that underwent significant changes (nine up-regulated and one down-regulated) were identified (Table 2). Of the 10 proteins, three have been previously reported to play a positive role in the inflammatory process: Haptoglobin, Alpha-1-antitrypsin, and Complement factor B, which supports our findings (Chou et al., 2012; Ostvik et al., 2014; Yang et al., 2017; Cholette et al., 2018; Balbi et al., 2019; Reeves et al., 2019). It is proposed that inflammation, originated from CP, might add to the inflammatory pool in the serum by contributing several pro-inflammatory mediators, such as C-reactive protein, interleukin (IL)-1, IL-6, and TNF-α, which cause cell apoptosis, tumor genesis, neuro-inflammation, and a systemic immune response (Rapone et al., 2019; Wang et al., 2019).

Recently, an association between CP and AD was hypothesized. It was suggested that CP worsened the inflammatory processes of AD in the brain. This was mainly attributed to increased levels of pro-inflammatory mediators, which activate the already primed microglial cells within the central nervous system and hasten the neuro-degeneration process (Wu and Nakanishi, 2015; Gil Montoya et al., 2020). In this study, of the 10 differentially expressed proteins, five have been extensively studied concerning the pathology of AD: Cofilin-2, Triosephosphate isomerase, Cathepsin B, Clusterin, and ITI-H4, which strengthens the possibility of an association between the two diseases. To further validate our results, these five proteins were analyzed by western blot. Cofilin-2, Cathepsin B, and Clusterin levels were increased and ITI-H4 levels were robustly reduced in the whole serum from CP patients. Although Triosephosphate isomerase was observed to be altered on 2D-DIGE, western blot detected no significant alteration. Of the five AD-related proteins above, Cathepsin B was chosen for further study with the highest correlation with the MMSE score (*r* = −0.874, *p* < 0.001).

Nowadays, as there is still no effective treatment or curable drugs for AD, early and accurate diagnosis has become a prime requirement for the management of AD. CSF diagnosis (by invasive means) may not always be feasible, and better non-invasive diagnostic techniques are needed. This is especially important since many AD patients are elder people, whose body is relatively poor and cannot bear the impact of spinal puncture. A large part of CSF proteins is draining out of the brain, which makes serum as a protein pool at the forefront for diagnosis. For Cathepsin B, our future study was to check if their quantity varies during aging and/or as periodontitis diagnosis timeline increases. Usually, it takes 10 years from diagnosis of periodontitis to become a risk factor for AD, so there could be a steady increase in Cathepsin B overtime after the initial diagnosis of periodontal disease. Cathepsin B could be a potential biomarker to make stronger associations with periodontitis and AD.

Evidence also indicates that Cathepsin B has β-secretase activity, participating in APP-processing and cutting to Aβ (Perlenfein and Murphy, 2017; Batkulwar et al., 2018; Sun et al., 2018). Therefore, the molecular mechanism of Cathepsin B in the pathology of AD is interesting. There are two sources of serum Cathepsin B, peripheral organs leaking and brain penetration. It has been reported that macrophages-produced Cathepsin B involves in peripheral Aβ production both *in vitro* and *in vivo*. Cathepsin B but not BACE1 could cut the wild-type β-secretase site effectively and further into Aβ_1–42_ and Aβ_3–42_ (Nie et al., 2019). In the central nervous system, Cathepsin B is mainly secreted by microglia and packaged into neuronal secretory vesicles with its signal peptide, which will either be captured by neurons or circulate into the CSF. Upon BBB damage, Cathepsin B is absorbed into the serum. Our results also showed at least part of the serum Cathepsin B comes from brain penetration (data not shown). Cathepsin B has been also confirmed to promote mature IL-lβ processing and secretion *via* activated microglia. Our results demonstrated that the protein level of Cathepsin B was significantly increased along with drastic augmentation in both Aβ_1–40_ and Aβ_1–42_ levels in SK-N-SH APPwt cells treated with 1 μg/ml P.g-LPS for 7 days. Furthermore, inhibition of Cathepsin B could result in a significant reduction in both the Aβ_1–40_ and the Aβ_1–42_ levels (Figures 4G,H). This is important because, despite evidence indicating the comorbidity of CP and AD, no studies demonstrate the causal link between CP and AD (Harding et al., 2017). Our study, for the first time, demonstrated that Cathepsin B might be a key link between the two diseases.

Clarified by several other studies, the pathology of CP is accompanied with the outbreak of inflammatory mediators, especially pro-inflammatory cytokines such as IL-6, IL-1β, TNF-α, Human NF-κB p65, and CRP (Singhrao et al., 2015; Teixeira et al., 2017; Ranjan et al., 2018; Singhrao and Olsen, 2018, 2019). Wu et al. (2017) found IL-1β, which was produced by microglia, could increase the level of Cathepsin B APP through IL-1R signaling in primary neurons after P.g-LPS treatment. IL-1β was the key inflammatory mediators that link CP and AD (Wu et al., 2017). Nevertheless, our results showed that only TNF-α treatment could significantly upregulate the protein level of Cathepsin B. TNF-α could be an intermediate between P.g-LPS treatment and the production of Cathepsin B and APP. These different results were probably due to the source of inflammatory mediators: microglia-derived IL-1β and exogenous TNF-α. Future microglia and primary neuron co-culture systems are needed to confirm the results of this study.

There are also arguments for this work. Our clinical serum study showed Aβ_1–42_/Aβ_1–40_ ratio was notably lower (*p* = 0.009) in the CP group, which is different from the previous report. Elevated plasma Aβ_1–42_/Aβ_1–40_ ratio was observed in severe periodontitis group than in other groups (moderate/mild/absent; Gil-Montoya et al., 2017). Those differences were most likely attributed to the source and degree of patients. Because after we separated the participants into severe, moderate, mild, and control, we also got similar results as what Gil-Montoya et al. (2017) have reported. However, only three CP patients were detected as severe periodontitis, which made the results of subgroup unreliable (date not shown). Anyway, it is well-known that lower serum Aβ_1–42_ level and Aβ_1–42_/Aβ_1–40_ ratio could be found in AD patients as compared to age-matched normal controls, which strongly supports our findings.

In conclusion, in our exploration study, 10 differential expressed proteins were identified between CP and Control groups, while, in the validation study, Cofilin-2, Cathepsin B, Clusterin, and ITI-H4 exhibited a significant alteration in the whole serum samples. Also, Cathepsin B might be a link between CP and AD. Our study represents a new hope for therapeutic interventions that could prevent the progression and worsening of AD.

## Data Availability Statement

The raw data supporting the conclusions of this article will be made available by the authors, without undue reservation.

## Ethics Statement

The studies involving human participants were reviewed and approved by Lian Zhang Medical Ethics Committee, Shenzhen Baoan Women’s and Children’s Hospital. The patients/participants provided their written informed consent to participate in this study.

## Author Contributions

XR finished 2D-dige and wrote the article. LX contributed to the *in vitro* study. YL contributed to cell culture. HY was in charge of sample collection. WC and LL contributed to western blot and ELISA. DL revised the article. XZ provided the idea and funding of the study.

## Conflict of Interest

The authors declare that the research was conducted in the absence of any commercial or financial relationships that could be construed as a potential conflict of interest.
