# Supplementary material for: Chronic Periodontitis and Alzheimer Disease: A Putative Link of Serum Proteins Identification by 2D-DIGE Proteomics
Source: Front Aging Neurosci. 2020 Aug 21;12:248. doi: 10.3389/fnagi.2020.00248 (PMC7472842; doi:10.3389/fnagi.2020.00248)
Supplement: Supplementary file 1 [file Data_Sheet_1.docx]

**Brief introduction of PD animal model construction**

When APP/PS1 mice was around 8 weeks, P.g-LPS (2ul, 1mg/ml)was injected into the palatal side of the first and second molars, and 4-0 silk thread was ligated on the second molars for 3 months.

**Figure S1: A apart of serum Cathepins B and Clusterin comes from CSF**


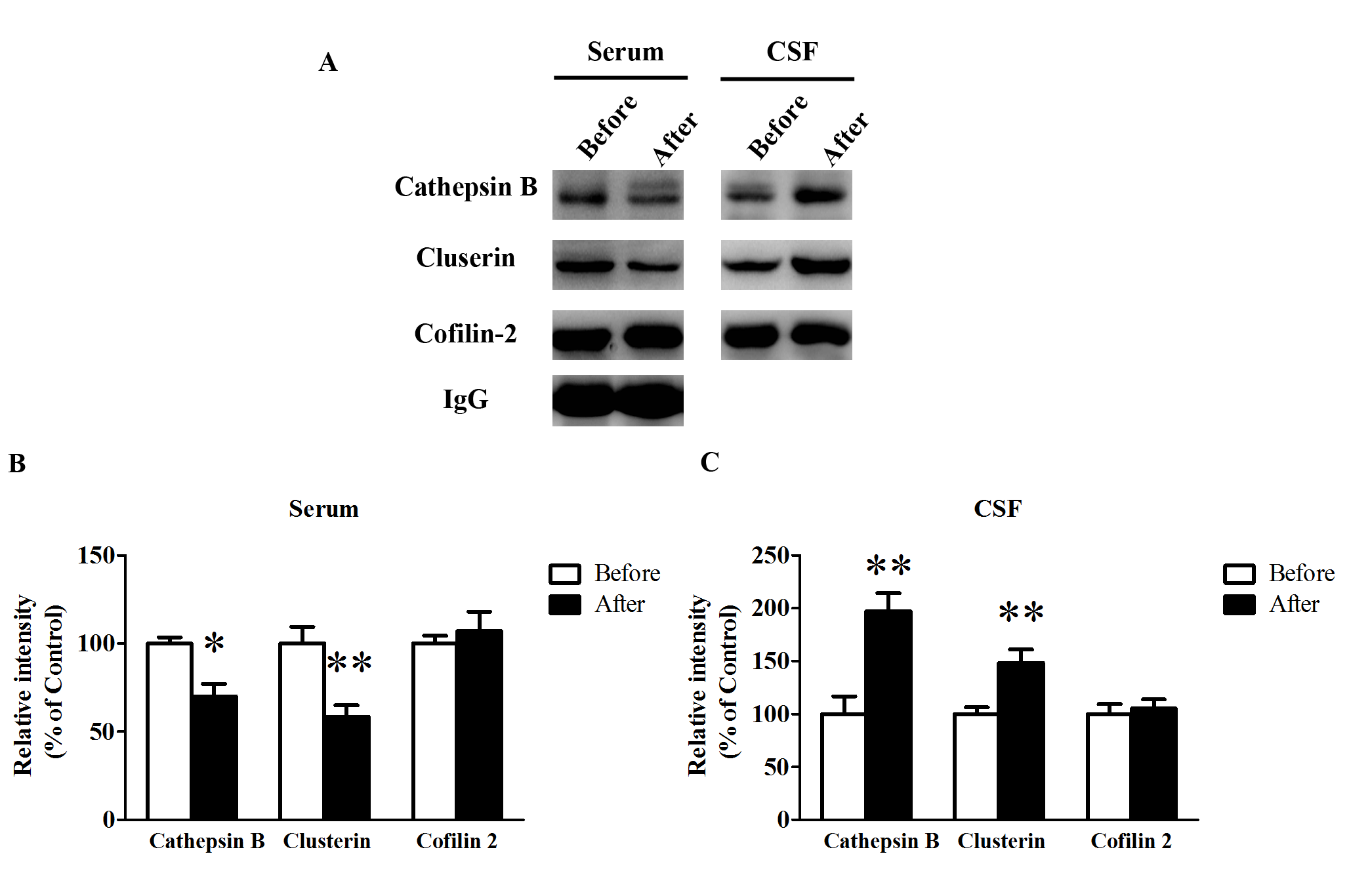


**Figure S1 The protein level of Cathepsin B, Clusterin and Cofilin 2 in the CSF and Serum in PD animal mice before after injection Wnt-S surrogate (*i.v.*)(n=4)**

Serum and CSF were collected 24h before Wnt-S surrogate injection. After 24h injection, Serum and CSF for the same mice were collected again. The volume of serum and CSF for each collection was 10ul.

**Figure S2: P.g-LPS receptor TLR4 can be detected in SK-N-SH cells APPwt cells**


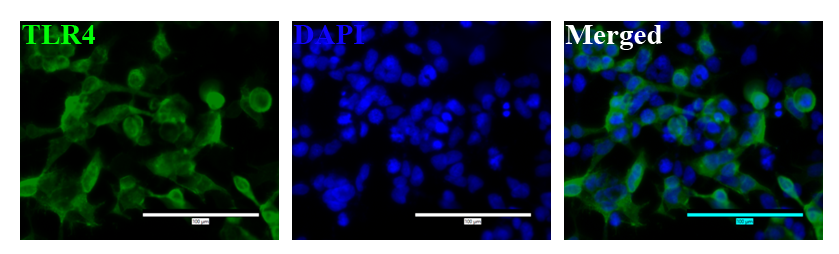


**Figure S2. The expression of TLR4 in SK-N-SH cells APPwt cells**

TLR4 (Green), nuclear (blue).

Anti-TLR4 antibody (Abcam, ab13556) dilution 1/100

The protocol of IF was described in *Rong et al, CNS Neurosci Ther.2017,*

**Figure S3**

**
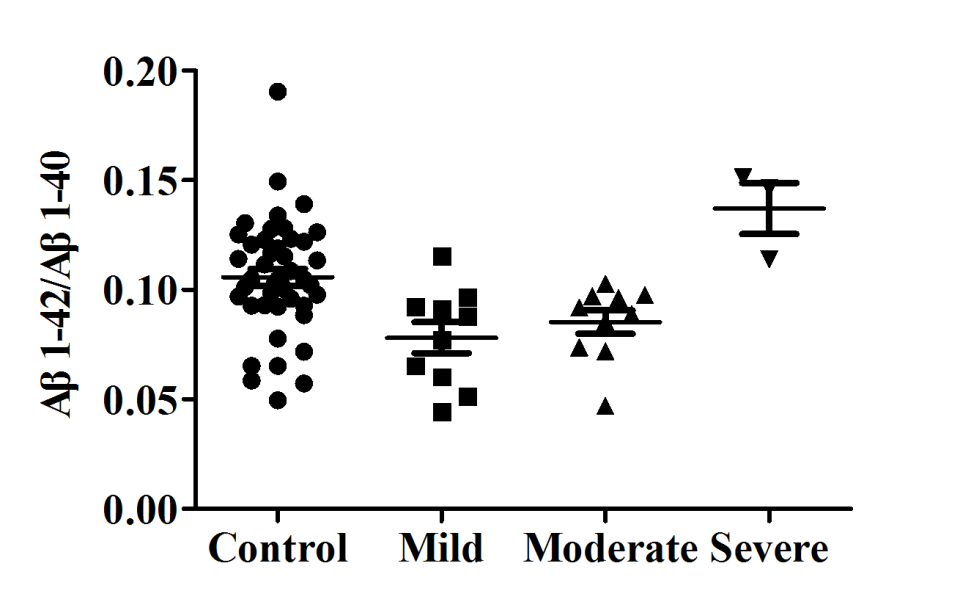
Aβ_1-42_/Aβ_1-40_ ratio in severe, moderate, mild periodontitis group and control group**

**Figure S3. Aβ_1-42_/Aβ_1-40_ ratio in severe, moderate, mild periodontitis group and control group.** There are also caveats for this subgroup, because only three CP patients were detected as severe periodontitis, which made the results of subgroup unreliable and that's why we did not subgroup the CP patients in our study.

**Table S1: MMSE of each participant.**

| Control | | | | | CP | | |
| --- | --- | --- | --- | --- | --- | --- | --- |
| C1：26 | C10：27 | C19：29 | C28：30 | C37：29 | CP1：24 | CP10：19 | CP19：24 |
| C2：29 | C11：30 | C20：26 | C29：27 | C38：30 | CP2：17 | CP11：20 | CP20：23 |
| C3：30 | C12：28 | C21：27 | C30：30 | C39：28 | CP3：16 | CP12：22 | CP21：15 |
| C4：27 | C13：27 | C22：29 | C31：29 | C40：27 | CP4：18 | CP13：22 | CP22：21 |
| C5：27 | C14：27 | C23：26 | C32：30 | C41：27 | CP5：23 | CP14：23 | CP23：19 |
| C6：30 | C15：29 | C24：26 | C33：26 | C42：28 | CP6：19 | CP15：24 |  |
| C7：26 | C16：26 | C25：30 | C34：27 | C43：29 | CP7：22 | CP16：20 |  |
| C8：30 | C17：26 | C26：27 | C35：26 | C44：26 | CP8：21 | CP17：20 |  |
| C9：28 | C18：29 | C27：29 | C36：30 | C45：26 | CP9：18 | CP18：19 |  |

**Table S1: Dr. Chen, the best nerve physician in our hospital, tested the MMSE for each patient.**
